# Integrating Astrocytes in the Sleep–Wake Cycle: The Time Is Now

**DOI:** 10.1002/bies.70077

**Published:** 2025-09-30

**Authors:** Marco Brancaccio

**Affiliations:** ^1^ Department of Brain Science Imperial College London London UK; ^2^ UK Dementia Research Institute at Imperial College London London UK

## Abstract

Astrocytes are emerging as critical regulators of the sleep–wake cycle, actively contributing to both sleep homeostasis and circadian rheostasis. This dual role challenges neuron‐centric frameworks that have dominated sleep and circadian biology and highlights astrocytes as potential integrators of internal temporal information. Experimental evidence shows that astrocytic calcium dynamics correlate with sleep state and that manipulating astrocytes can alter sleep architecture and homeostasis. In parallel, key aspects of circadian timekeeping can be autonomously driven by astrocytic clocks, with pulses of rhythmic GABA and glutamate able to synchronize circadian circuits and support circadian patterns of behavior. These findings are coherent with the idea that astrocytes can act as context‐dependent integrators to convey environmental cues and internal states to neuronal circuitries and promote adaptive behavior. Incorporating astrocytes into conceptual models of the sleep–wake cycle may help reconcile contradictory findings and offer new frameworks to better understand how salient internal temporal representations are encoded within the brain.

## Graphical abstract

Astrocytes actively regulate sleep homeostatic and circadian rheostatic processes, underpinning the sleep–wake cycle. By acting as context‐dependent integrators of internal state and rhythmic environmental inputs, they modulate the encoding properties of neuronal circuitries and contribute to salient temporal representations in the brain.

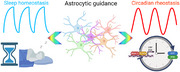

**Abbreviations**
BMAL1: basic helix‐loop‐helix ARNT‐like protein 1
CLOCK: circadian locomotor output cycles kaput
CRY: cryptochrome
GABA: gamma‐aminobutyric acid
GAT3: GABA transporter 3
GPCR: G protein‐coupled receptors
IP3R2: inositol 1,4,5‐trisphosphate receptor 2
NMDA: N‐methyl‐D‐aspartate
NREM: non‐REM
PER: period
REM: rapid eye movement
STIM1: stromal interaction molecule

## 1 Introduction

Homeostasis, the key process by which living organisms maintain a stable internal state despite external environmental fluctuations, is a well‐recognized cornerstone of physiological stability [1]. It is essential for survival, as physiological systems must operate within narrow limits to function optimally. Homeostasis regulates various aspects of metabolism (e.g., body temperature, blood pH, glucose levels) and behavior (e.g., sleep, feeding). Negative feedback is the most common mechanism underpinning homeostasis, working to counteract deviations from an endogenously determined setpoint. Homeostasis is, therefore, critically dependent on these setpoints—internally defined values that optimize biological responses to immediate environmental changes. Such setpoints are, however, not fixed and change in time to meet predictable changes of the environment, a process known as (programmed) rheostasis (*rheos*: change; *stasis*: stability) [2, 3]. As opposed to homeostasis, rheostasis is rooted in feedforward mechanisms of genetic origin, preparing organisms to meet environmental variations: it mediates an anticipatory, rather than reactive, form of *stasis* (i.e., equilibrium) with the environment. Rheostasis is critically important for the broader adaptability of species to their biological niches and, ultimately, their endurance. Examples of biological rheostasis are widespread and include seasonal behaviors such as hibernation and migration [3]. However, the most predominant environmental cycle, to which most species must adapt to survive and thrive, is the 24 h cycle determined by the Earth's rotation.

The sleep–wake cycle is a key example of how a complex behavior may emerge from a finely orchestrated interplay between homeostatic and rheostatic processes. Prevailing conceptual frameworks such as the two‐process model [4] indeed recognize the contributions of these two processes (Process C: rheostasis; Process S: homeostasis) to sleep regulation. Such models, which have driven very successful experimentation over the last 40 years, are neuronocentric and consider such contributions to be functionally and mechanistically distinct.

I will discuss recent reports suggesting that astrocytes may play a critical role in both the sleep homeostasis and circadian rheostasis processes underpinning sleep–wake regulation. These may serve as a testbed for future investigations aimed at validating or challenging the role of astrocytes as key players of the sleep–wake cycle. I will then propose how including astrocytes as cell integrators of the sleep–wake cycle may help produce more accurate theoretical generalizations about the sleep–wake cycle, both in physiological conditions and in response to brain insults. Such a theoretical framework may als aid future investigations about the role of other glial cell types involved in the regulation of circadian and sleep function, which are beyond the scope of this essay (Box 1).

**BOX 1: Looking beyond the stars: Microglia and oligodendrocytes in sleep and circadian rhythms**

Understanding how circadian activities in other glial cell types are coordinated to warrant robust sleep–wake cycles intertwined with rhythms of immunity and nerve repair is an area of fervent research activity. Astrocytes are not the only non‐neuronal cells that have been recently shown to be involved in the regulation of sleep and circadian rhythms. Microglia display pronounced circadian rhythmicity in morphology and gene expression, regulated in part by core clock genes [48, 49]. Their surveillance and synaptic pruning activities fluctuate across the sleep–wake cycle, with sleep favoring microglial processes that stabilize synapses, while wakefulness enhances pruning and pro‐inflammatory signatures [50]. Perturbation of microglial clocks impairs sleep architecture and alters responses to sleep loss, implicating them as dynamic modulators of synaptic homeostasis and circadian resilience [51]. Oligodendrocytes, traditionally associated with myelin production, also exhibit sleep–wake‐dependent dynamics. Transcriptomic profiling shows that genes involved in lipid and myelin synthesis are preferentially upregulated during sleep, whereas those linked to cell proliferation dominate during wakefulness [52]. Oligodendrocyte precursor cells (OPCs) and their downstream derivatives, oligodendrocytes, possess intrinsic molecular clocks. Key clock genes—Bmal1, Per2, and Rev‐Erbα—show rhythmic expression in OPCs; disruption of Bmal1 in these cells diminishes OPC proliferation, alters morphology, and leads to thinner myelin, cognitive and motor deficits, and sleep fragmentation [53]. Sleep appears essential for OPC proliferation and differentiation, processes that are disrupted by chronic sleep deprivation [54]. Although less is known about intrinsic circadian oscillations in oligodendrocytes, emerging evidence suggests they express functional clocks and interact with astrocytic and neuronal rhythms to influence white‐matter plasticity. Together, these findings highlight a potential role for other glial cell types in circadian and sleep regulation: microglia may tune synaptic networks and regulate pro‐ versus anti‐inflammatory immune responses in a time‐of‐day‐dependent manner [55], while oligodendrocytes may couple myelin plasticity to the sleep–wake cycle [56].

## 2 Astrocytes as Active Players in Sleep Homeostasis

In 1895, Cajal hypothesized that astrocytic processes invade the synaptic cleft during sleep, acting as a circuit breaker, while their retraction would signal the transition to wakefulness [5]. However, the key conceptual question of whether and how astrocytes may actively participate in changes in brain state associated with the sleep–wake cycle has been evaded until recently. While Cajal's original hypothesis of sleep state‐dependent variation of astrocytic synaptic coverage has found experimental confirmation in ultrastructural studies in cortical areas, the variations in synaptic coverage are opposite to what he expected: astrocytic processes get closer to synapses during wake [6]. A proposed physiological explanation is that during wakefulness increased synaptic coverage would more efficiently clear glutamate from the synaptic cleft (astrocytes express high‐affinity glutamate transporters), while reduced coverage during sleep would allow for glutamate spillover and transsynaptic synchronization underpinning highly coherent activity during NREM sleep [6]. While retraction of astrocyte processes during sleep is consistent with studies showing increased interstitial space volume driven by astrocytically expressed aquaporins (i.e., AQP4) [7], whether this enlarged interstitial volume may be associated with increased or reduced clearance of brain catabolites during sleep is an area of intense debate [8]. Experiments of sleep deprivation in rodents, a common paradigm to interrogate mechanisms of sleep homeostasis, have not yet resolved the matter: while sleep deprivation reduces interstitial space volume and reduces astrocyte clearance, it also increases synaptic coverage by astrocytes to boost clearance [6]. Moreover, MRI studies of sleep deprivation in humans did not find it to be associated with increased interstitial brain volume [9]. Future studies combining high‐resolution imaging of the interstitial space alongside probes monitoring extracellular glutamate [10] and other key neuromodulators linking neuronal activity to astrocyte metabolic sensing, such as adenosine [11, 12] across the sleep–wake cycle at the synapse level, may provide a platform to disentangle the matter. Combining such an approach with ad hoc molecular manipulations of AQP4 expression/activity may help further clarify mutual causal relationships. Cross‐species comparisons, particularly integrating rodent and human data via multimodal MRI and PET imaging, may also help reconcile divergent findings. Finally, computational modeling of glymphatic flow dynamics under varying astrocytic configurations could provide mechanistic insights and guide experimental design.

These apparent inconsistencies highlight a fundamental problem in establishing causal connections between astrocyte activity and sleep homeostasis: the lack of a widely accepted and translatable readout/proxy to measure astrocyte activity during sleep. Astrocytic calcium dynamics directly correlate with both sleep state and homeostasis and may serve as a more consistent proxy for the astrocyte involvement in sleep homeostasis. Real‐time in vivo monitoring by photometry/miniaturized microscopy of intracellular astrocytic calcium by genetically encoded calcium indicators (GECIs) in animal models offers some promise. Higher levels of astrocytic calcium are associated with wakefulness in upper brain areas (e.g., cortex, hippocampus), with a sharp decrease during sleep [13, 14, 15, 16]. Sleep deprivation increases the amplitude and duration of astrocytic calcium transients, more prominently so in distal astrocytic processes closely associated with synapses, and scales proportionally with sleep pressure during the NREM phase [14]. Interestingly, changes in intracellular calcium levels precede EEG variations, both in the transition from NREM to wakefulness and vice versa, thus suggesting that astrocytes are not merely reacting to changes in neuronal activity [13, 17]. Astrocytic ablation of molecular transporters contributing to calcium influx to the ER, such as STIM1, attenuates homeostatic sleep responses without altering general baseline sleep architecture [14]. Ablation of the astrocytic‐enriched IP3R2 [18], mediating the calcium efflux from the ER [19], leads to fragmented sleep and diminished delta power during NREM sleep [13]. Similarly, designer chemogenetic manipulations regulating intracellular calcium levels by GPCR produce selective and distinct effects on sleep. In particular, G_i_ stimulation increases the frequency of intracellular calcium transients and increases slow wave sleep activity during the NREM phase, whereas G_q_ activation prolongs NREM sleep but does not affect slow wave sleep [20]. G_q_ stimulation in hippocampal astrocytes decreases wakefulness time and increases NREM and REM sleep time, without affecting cortical oscillations, whereas stimulation of pontine astrocytes suppresses REM sleep and enhances delta power during NREM sleep, thus revealing brain area‐specific effects of G_q_ pathway stimulation on sleep function [21]. Some caution should be exercised when interpreting these findings: (i) ablating endogenous pathways regulating calcium dynamics is likely to interfere more broadly with aspects of cellular physiology related to calcium equilibrium; (ii) designer manipulations can drive calcium levels beyond a physiologically meaningful range. Nevertheless, diverse experimental approaches now point to an active role for astrocytes in the regulation of sleep and its homeostasis and to intracellular calcium as a marker for sleep‐associated astrocytic activities, as well as an entry point to interrogate the role of astrocytes in sleep homeostasis.

## 3 Astrocytes as Active Players in Circadian Rheostasis

While the role of astrocytes in modulating sleep homeostasis is supported by several recent findings, it is still unclear to what degree this modulation may amount to autonomously determining specific aspects of sleep homeostasis. As opposed to the highly distributed circuit organization and poorly defined genetic determinants underpinning sleep homeostasis, circadian rheostasis is supported in mammals by highly hierarchical structures, both in terms of their intracellular molecular and intercellular network organization [22, 23]. This has allowed testing more directly whether astrocytes can cell‐autonomously support specific aspects of circadian rheostasis [24, 25].

Briefly, specialized clock genes pivot in self‐sustained cycles of roughly 24 h (circa‐about; diem‐daily) whereby dimers formed of the core clock proteins CLOCK and BMAL1 peak during daytime and promote transcription of negative clock regulators Per and Cry. Nighttime accumulation of PER/CRY dimers progressively inhibits BMAL1/CLOCK‐mediated transcription of Per and Cry (a mechanism known as the core transcription‐translation feedback loop, TTFL), until degradation of PER/CRY signals the beginning of a new circadian cycle. While such a core molecular clockwork is virtually present in all cells and tissues, body‐wide synchronization and alignment to the light–dark cycle critically depend on a central timepiece, the suprachiasmatic nucleus (SCN) of the anterior hypothalamus. The SCN can specify stable sleep–wake cycles even in temporal isolation (i.e., no access to environmental light), as well as entrain daily physiology to the light–dark cycle, thanks to the input it receives from the retina [22, 23]. Several investigations have shown that astrocytes can actively specify aspects of circadian rheostasis. In particular, (i) ablating Bmal1 in SCN astrocytes lengthens the circadian period [26], (ii) altering TTFL periodicity in SCN astrocytes predictably modulates rest‐activity behavior [24, 26], and, critically, (iii) expressing Cry in SCN astrocytes in genetically arrhythmic mice (double Cry1/2‐null mice) is sufficient to specify stable sleep–wake cycles [25]. Notably, neurons undergoing equivalent experimental manipulations also change the circadian periodicity of the animal and support stable sleep–wake cycles [24, 25], thus suggesting that neuronal and astrocytic activities in the SCN are mutually reinforcing in physiological conditions. However, the periodicity of the emerging sleep–wake cycles in Cry‐rescued animals is inversely related to the numbers of targeted neurons and astrocytes, respectively (higher numbers of targeted neurons—longer periodicity; higher numbers of targeted astrocytes—shorter periodicity), thus suggesting that a complex astrocyte‐neuronal “pushing and pulling” period interplay may underpin circadian rheostasis [25]. In fact, astrocyte intracellular calcium, monitored by GECI in SCN explants, peaks during nighttime, while simultaneously detected neuronal calcium peaks during daytime [24, 27]. This striking time separation in the daily activation of astrocytes and neurons is not limited to intracellular calcium but also extends to the TTFL, as shown by the distinct phases of Cry1 transcription monitored by clock gene reporters expressed in neurons or astrocytes, respectively [25]. The emerging picture of daytime‐active SCN neurons and nighttime‐active astrocytes further suggests that an astrocyte‐neuronal interplay may be at the heart of circadian rheostasis in mammals.

## 4 Integrating Astrocytes in the Sleep–Wake Cycle

The establishment of a causal link from astrocyte clock genes to SCN circuit activities to sleep–wake behavior provides a framework to investigate the nature of the information encoded by astrocytes and their specific contributions to the sleep–wake cycle. Moreover, it offers opportunities to gain fresh perspectives on seemingly inconsistent/contrasting evidence, which are difficult to reconcile within a purely neuronocentric view of the sleep–wake cycle.

One example is understanding how SCN neurons, which are mostly GABAergic [28] (> 95%), would support self‐sustaining circadian cycles of activity, even when isolated in organotypic cultures for several weeks [29]. A purely inhibitory GABAergic circuit would be expected to progressively dampen activity over time. The role of GABA transmission in the SCN has puzzled chronobiologists for the last 30 years, with GABA being reported to be mostly inhibitory, partially excitatory, or both inhibitory and excitatory depending on circadian phase (for a recent review, see [30]). Daily addition of GABA can indeed synchronize SCN neurons [31], but inhibiting GABA receptors does not reduce SCN synchronization; it enhances it [32, 33]. While GABA is the only classical neurotransmitter expressed in the SCN, neither the ablation of key enzymes involved in GABA production (i.e., glutamate decarboxylases GAD65/67), nor the vesicular GABA transporters, nor the inhibition of GABA_A/B_ receptors significantly affects SCN circadian timekeeping [32, 33].

To add to the puzzle, while SCN neurons are more active during the day, GABA levels in the SCN peak at nighttime [29, 34]. Circadian oscillations of GABA with a nighttime peak have indeed been recently confirmed by using genetically encoded live imaging fluorescent GABA probes [27, 35, 36]. This timing is as predicted by previous working models: release of glutamate during nighttime by SCN astrocytes would promote GABA release in the synaptic cleft via stimulation of presynaptic NMDA receptors containing the NR2C subunit [22, 23, 24].

**FIGURE 1:**
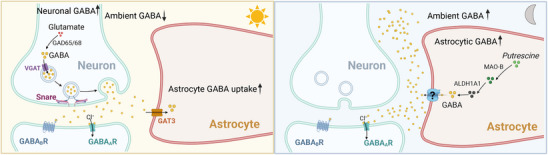
A model for cell‐autonomous astrocytic circadian regulation of ambient GABA in the suprachiasmatic nucleus. In the suprachiasmatic nucleus (SCN), GABA is produced from glutamate degradation in neurons during daytime, and from polyamines (e.g., putrescine) in astrocytes during nighttime. Conversely, uptake of synaptic GABA is upregulated during daytime by increased expression of the GABA transporter GAT3 expressed in astrocytes, and downregulated during nighttime. Astrocytes are therefore primarily responsible for the generation of circadian oscillations of ambient GABA reported in the SCN. This model is based on current experimental evidence [27, 36].

However, more recent investigations have revealed that astrocytes may play a more direct role in creating GABA rhythms in the SCN: inhibiting GABAergic transmission by blocking synaptic vesicle fusion in the SCN does not significantly affect GABA rhythms, even if it dampens and desynchronizes circadian rhythms of neuronal calcium and clock gene expression [27]. In contrast, pharmacological inhibition of biosynthetic pathways producing GABA from polyamines selectively expressed in SCN astrocytes diminishes or abolishes circadian GABA oscillations [27, 37]. Moreover, inhibiting astrocytically expressed GABA transporters (GAT3) leads to GABA accumulation and dampens circadian oscillations of neuronal calcium and clock gene expression [36]. A coherent picture emerges when live imaging experiments, pharmacological manipulations, and gene expression studies are considered together: GABA is produced by astrocytes at night (from polyamines, which may explain the inefficacy of GAD65/67 ablation in altering GABA rhythms) and cleared during the day, thus autonomously creating ambient circadian GABA rhythms within the SCN [27, 36, 38] (Figure 1). Blocking synaptic GABA transmission desynchronizes neuronal rhythms and clock gene expression but does not affect GABA rhythms, thus confirming this autonomy [27]. Neuronal and astrocytic circadian oscillations can be experimentally separated; thus, they are not fully interdependent.

In keeping with such a separation, the spatiotemporal organization of astrocytic and neuronal activation within the SCN are distinct, with neurons expressing dorsomedial to ventrolateral daily waves of activity with a range of 2–3 h, while astrocytes express a quasi‐homogeneous phase, as reported by several readouts, including GABA [27]. A variable phase relationship between neurons and astrocytes thus exists along the circadian cycle in the SCN, suggesting that partially parallel streams of information can exist within the SCN and may be enshrined in astrocytes and neurons, respectively. This also implies that astrocytes may be potentially “blind” to specific aspects of circadian neuronal regulation, such as phase, and explains their inability to affect it [39]. Similarly, GABA rhythms can be maintained even if the neuronal distribution of phases within the SCN is vastly reduced by blocking GABAergic synaptic transmission [27]. Astrocytes may thus contribute to accurate circadian periodicity in the SCN circuit by autonomously releasing synchronizing rheostatic pulses of GABA and glutamate in the SCN ambient (internal astrocytic synchronizers—“astrozeits” [27]), while neurons will be uniquely responsible for the timed engagement of downstream targets driving rhythmic behavior.

Could this insensitivity to neuronal phase be physiologically relevant for entrainment, in addition to the accuracy of timekeeping? Astrocyte processes differentially wrap around retinorecipient VIP neurons in the SCN during the light–dark cycle [40, 41]. This may allow astrocytes to modulate circadian gating by time‐regulating the sensitivity of the SCN circuit to the retinal input. Isolating astrocytes from the SCN neuronal phase may allow them to provide a more resilient and faithful internal representation of entraining cycles, thus enhancing realignment of circadian physiology to the light–dark cycle.

## 5 Astrocytes as Context‐Dependent Integrators of the Sleep–Wake Cycle

**FIGURE 2:**
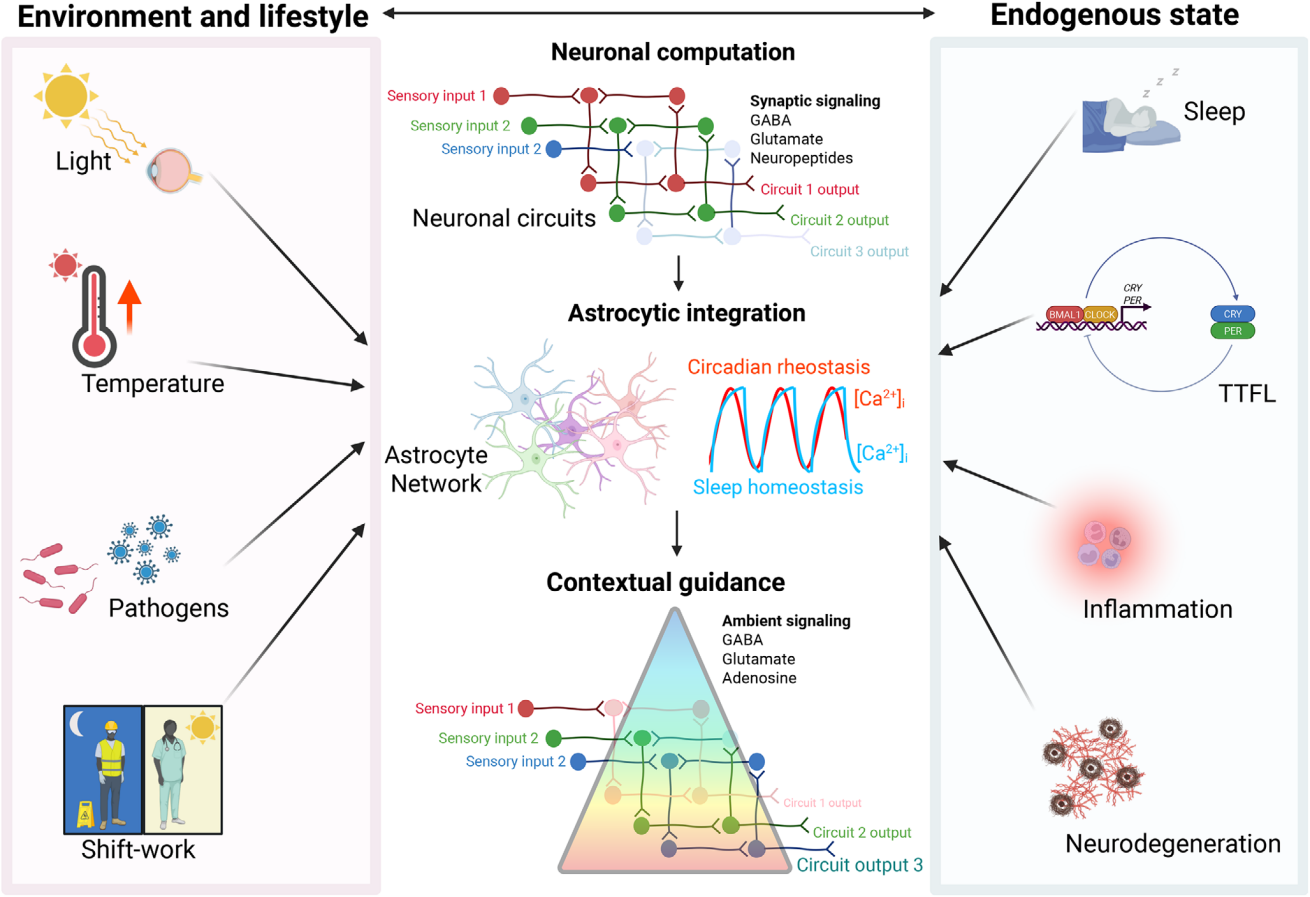
Astrocytes provide contextual guidance to circuits regulating the sleep–wake cycles. According to the contextual guidance framework, astrocytes can directly sense changes in the internal state, triggered by exogenous (e.g., environment and lifestyle), and endogenous factors. Astrocytes can integrate over time signals connected to the internal state (e.g., noradrenaline, cortisol, and inflammatory cytokines) to directly modulate the activity of several circuitries responsible for circadian and sleep behavior.

How can the role played by astrocytes in sleep homeostasis and circadian rheostasis be conceptualized within their wider role in regulating complex behavior? A recent framework (contextual guidance) identifies astrocytes as state‐dependent orchestrators of neuronal circuits [42] (Figure 2). In prevailing models, sensory input first triggers neuronal responses, which may then recruit astrocytes that, in turn, modulate circuit responses. In contextual guidance, astrocytes provide a parallel upstream input to neurons, directly integrating and conveying *(computation)* salient information about the external and internal environment *(context)* to gate *(trigger)* meaningful adaptive behavioral responses *(adaptation)*. In line with these views, astrocytes are active during the circadian night *(context)*, expressing clock gene oscillations that are sufficient to *trigger* highly synchronous rheostatic circadian pulses of GABA and glutamate *(computation)*, which specify spatiotemporally organized neuronal activities and rest‐activity behavior *(adaptation)* [25]. In sleep homeostasis, elevated astrocyte activity (as measured by calcium) is associated with wakefulness *(context)*; wakefulness progressively *triggers* intracellular calcium accumulation in astrocytes *(computation)* until it is homeostatically discharged with sleep *(adaptation)* [13, 14, 17]. Therefore, astrocytes can act as state‐dependent integrators for both circadian rheostasis and homeostasis.

## 6 Conclusions and Future Outlook

Current conceptualizations of the sleep–wake cycles (two‐process model) consider sleep homeostasis and circadian rheostasis as largely functionally and mechanistically distinct, notwithstanding more recent evidence highlighting a deeper entanglement than originally proposed [43, 44]. This entanglement does not, however, consider that some aspects of the sleep–wake cycle may be integrated by specific cell types, namely astrocytes. Astrocytes could integrate both circadian rheostasis (Process C) and sleep homeostasis (Process S) by using a shared messenger system (intracellular calcium) and may act as a convergence point for coregulation. Consistent with this, stable (non‐entraining) environmental temperature changes can tune the periodicity of circadian oscillations of neuronal calcium and clock‐controlled gene expression (but not clock gene expression) in the SCN. Moreover, temperature sensing in the SCN is dependent on a full Bmal1 complement [45]. As brain temperature is under strict homeostatic and circadian regulation, these data suggest that an intimate crosstalk between the two may indeed happen and potentially involve astrocytes. Investigating astrocytic circadian and homeostatic responses to changes in lifestyle (e.g., shift‐working, sleep deprivation) and disease‐related (e.g., neurodegenerative pathology, infection) [46, 47] disruptions will be critically important to understand how circadian misalignment arises and whether astrocytes integrate circadian rheostasis and sleep homeostasis. In response to inflammatory and immune responses, astrocytes undergo phenotypic changes that alter the extracellular milieu; this, in turn, may change the computations of the neuronal circuitries responsible for sleep–wake behavior, following the contextual guidance framework. It is therefore time to include astrocytes in our theoretical landscape when building models that more accurately describe how salient internal temporal information is encoded within the brain and how it may degrade in disease.

## Author Contributions

M.B. has written and edited the article and the figures, edit it, and aquired the funding.

## Conflicts of Interest

The author declares no conflicts of interest.
